# *In vitro* selection and optimization of high-affinity aptamer for milk allergen α-lactalbumin and its application in dual-mode detection

**DOI:** 10.3389/fnut.2022.1005230

**Published:** 2022-10-04

**Authors:** Ruobing Liu, Fuyuan Zhang, Minghui Shi, Yaxin Sang, Xianghong Wang

**Affiliations:** College of Food Science and Technology, Hebei Agricultural University, Baoding, China

**Keywords:** α-lactalbumin, aptamer, molecular docking, allergen detection, aptasensor

## Abstract

Milk is one of the most common sources of protein in people’s daily lives, and it is also recognized by the World Health Organization (WHO) as one of the eight categories of food allergies to human beings. α-lactalbumin (α-La) is the main cause of milk allergy. In this study, a single-stranded DNA aptamer with high binding affinity to α-La were selected using systematic evolution of ligands by exponential enrichment (SELEX) method. Compared with the full-length sequence, the binding affinity of the truncated aptamer LA-1t for α-La was increased six times using fluorescence analysis. Circular dichroism (CD) indicated that the secondary structure of LA-1t contained a typical hairpin structure. Through the docking simulation of LA-1t and α-La, these experimental results were further explained theoretically, and the recognition mechanism was explained. Finally, the colorimetric and fluorescence signal of boron nitride quantum dots anchored to porous CeO_2_ nanorods (BNQDs/CeO_2_) were modulated by FAM-labeled LA-1t to achieve highly selective and sensitive determination of α-La. This dual-mode sensing strategy displayed sensitive recognition for α-La in a linear range of 5–4,000 ng/ml with the LOD was 3.32 ng/ml (colorimetry) and 0.71 ng/ml (fluorescence), respectively. Simultaneously, the colorimetry/fluorescence dual-mode sensing strategy was applied for detecting α-La in spiked real samples and demonstrated good stability and reliability.

## Introduction

For Food allergy (FA) can be defined as the abnormal immune system reaction after eating a certain allergenic food (1). Following exposure to an allergen, susceptible individuals typically experience a range of symptoms including gastrointestinal distress, respiratory distress, hives, and systemic anaphylaxis (2, 3). In 1995, the Food and Agriculture Organization (FAO) divides allergenic foods into eight groups: milk, eggs, peanuts, tree nuts, wheat, soy, fish, and shellfish (4, 5). Cow’s milk allergy (CMA) is one of the most common food allergies, especially during childhood (6, 7). The World Health Organization (WHO), the U.S. Food and Drug Administration (FDA), and other food safety management organizations are contributing in collaborative work for standardization of detection approaches for allergens and also to establish requirements for food labeling. The assessment of allergen labeling demands development of rapid, accurate, and cheap analytical techniques for sensing and quantification of milk allergens, and the development of proper and reliable food labeling in order to ensure the life quality of allergic persons. Casein being present in the solid part and whey proteins present in the liquid part of the milk is thought to be the main causes of allergic reactions (8). α-Lactalbumin (α-La) is one of the main allergens in humans. α-La is often used as a nutritional protein because of its rich amino acid content, and it is also an important allergen. Studies conducted elsewhere in the world show that among patients with cow’s milk allergy, about 27.6–62.8% are allergic to α-La (9, 10). The allergenic potential of α-La is due to the presence of large amounts of epitopes, which selectivity bind IgE (11). The development of novel biosensing mechanisms for milk allergens α-La detection is imperative from the perspective of enforcement of labeling regulations and directives keeping in view the sensitive individuals. Research efforts have employed various techniques to detect α-La, including an enzyme-linked immunosorbent assay (ELISA) (12), Surface Plasmon Resonance (SPR) (13), electrochemical sensor (14, 15), Polymerase Chain Reaction (PCR) (16, 17), and chromatographic methods (18, 19).

Nucleic acid aptamers are single-stranded DNA (ssDNA) or RNA that can specifically bind to target molecules with high affinity, screened from chemically synthesized random oligonucleotide libraries by the Systematic Evolution of Ligands by Exponential Enrichment (SELEX) method. A wide range of targets can be identified by aptamers, including proteins, small molecules, cells, bacteria, peptides, and even tissues. Despite the presence of antibodies, aptamers can be generated by *in vitro* chemical processes with small batch-to-batch variation, high stability, and ease of chemical modification and label retention of activity unaffected (20, 21). Among food allergens detectable by apta-sensors, only a few examples of apta-sensors are reported in the literature to detect different milk allergens (22, 23), and there is no report on the aptamer for α-La.

Nanozymes are used to describe nanoscale materials with certain enzyme-mimicking properties (24). Compared with natural enzymes, nanozymes have significant advantages in terms of better stability, economy, and easy storage (25). To date, many nanomaterials with enzyme-mimicking properties have been discovered that can catalyze various chromogenic substrates to produce colored products in the presence of H_2_O_2_ (26, 27). Ce, as an important rare earth metal, has been further engineered into various nanomaterials with peroxidase-like activity due to its excellent redox properties. Meanwhile, CeO_2_ can quench the fluorescence signal through fluorescence resonance energy transfer (FRET) (28). Therefore, CeO_2_ nanomaterials can be applied in the construction of dual-mode sensing strategies, while retaining the advantages of low cost and simple operation of two different methods, while avoiding its inherent limitations that a single method may suffer in practical applications.

In this work, high-affinity aptamers against α-La were selected by capture-SELEX. Through the analysis of the consensus sequence and secondary structure, truncation optimization was carried out. It emerged that the LA-1t optimized by removing the redundant sequence in fact had a higher affinity for α-La. The secondary structure of LA-1t was characterized by prediction and circular dichroism (CD). Molecular docking analysis of LA-1t and α-La was done to predict possible binding sites and their interactions. Finally, based on the colorimetry/fluorescence dual-mode sensing strategy of aptamer-regulated boron nitride quantum dot-anchored porous CeO_2_ nanorods (BNQDs/CeO_2_), we achieved highly selective and highly sensitive detection of α-La.

## Materials and methods

### Materials and reagents

The initial ssDNA library for SELEX contained a central randomized region of 40 nucleotides (nt) flanked by: 20-nt constant region (5′-AGCAGCACAGAGGTCAGATG-40 random base -CCTATGCGTGCTACCGTGAA-3′); the biotin-labeled capture strand (C1: 5′-Biotin-AGCACGCATAGG-3′); the forward and reverse primers (F:5′-AGCAGCACAGAGGTCAGATG-3′; R:5′-P-TTCACGGTAGCACGCATAGG-3′) used for PCR; and all others unmodified, modified ssDNAs sequences were synthesized by Sangon Biotech Co., Ltd. (Shanghai, China). α-La, β-Lactoglobulin (β-Lg), casein (Cas) from bovine milk, bovine serum albumin (BSA), Immunoglobulin G from human serum (IgG) purchased from Sigma-Aldrich Co., Ltd. (USA). All PCR reagents and other electrophoresis compounds were purchased from Kangwei Biotech Co., Ltd. (Beijing, China). Streptavidin magnetic beads were obtained from BioMag Biotech Co., Ltd. (Wuxi, China). Graphene oxide (GO) was purchased from Guangzhou Wenrui Scientific Instrument Co., Ltd. (Guangzhou, China). The lambda exonuclease and 10 × lambda exonuclease reaction buffer was sourced from New England Biolabs Ltd. (Hitchin, UK). The other regular reagents were bought from Sinopharm Chemical Reagent Co., Ltd. (Shanghai, China).

### Systematic evolution of ligands by exponential enrichment procedure

Capture-SELEX performs aptamer screening by immobilizing oligonucleotide libraries. For coupling of ssDNA library to magnetic, a capture strand (C1) was designed to capture ssDNA library and couple streptavidin magnetic beads. The ssDNA library and C1 was dissolved and blended thoroughly in a molar ratio of 1:1.5 in binding buffer (BB: 50 mM Tris-HCl, 5 mM KCl, 100 mM NaCl, 1 mM MgCl_2_, and pH = 7.4). The mixture was heated at 95^°^C for 10 min and allowed to cool naturally to room temperature to allow complete complementary hybridization of the two components. Then, streptavidin magnetic beads with a mass ratio of 300:1 to the biotin-labeled complementary product were incubated at 37^°^C for 1 h. After the reaction, unbound sequences were removed by an applied magnetic field and washed three times with BB. Specifically, 1 nmol (100 pmol in the subsequent rounds) ssDNA library was immobilized and bound by incubation with α-La in binding buffer at 37^°^C on a shaker. As selection progressed, the time was reduced to 45 min and the α-La concentration was reduced to 30 μg/ml. Then, the supernatant was collected by magnetic separation and used as a template for PCR amplification. The PCR reaction was performed in 50 μL reaction buffer containing 2 μL ssDNA template, 1 μL forward primer (20 μM), 1 μL reverse primer (20 μM), 25 μL Mix, and 21 μL ddH2O.

Polymerase chain reaction conditions were as follows: 95^°^C predenaturation for 5 min, followed by optimized cycles of denaturation at 95^°^C for 30 s, annealing at 58^°^C for 30 s, extension at 72^°^C for 30 s, and a final extension step of 5 min at 72^°^C, and 4% agarose gel electrophoresis and imaging were used to identify PCR products. The next round of ssDNA libraries was prepared by specific digestion with lambda exonuclease at 37^°^C for 1 h. After being completely digested into ssDNA, the digestion mixtures were heated to 75^°^C for 10 min to inactivate the enzyme. The digestion products were tracked by 8% denatured polyacrylamide gel electrophoresis. Finally, the ssDNA was purified by a phenol chloroform method, quantified using a Nano Drop K5500 spectrophotometer, and served as a nascent ssDNA pool for the next round of SELEX.

The sixth, eighth, and tenth rounds introduce negative screening. After immobilizing the ssDNA library prepared in the previous round on streptavidin magnetic beads, it was first incubated with β-Lg, Cas, BSA, and IgG (total amount 50 μg/ml). Subsequently, the supernatant was discarded and 50 μg/ml α-La was added to the beads for positive selection.

### High-throughput sequencing

The ssDNA enriched in the last round was PCR amplified using unmodified primers. The PCR products were subjected to high-throughput sequencing at Sangon Biotech Co., Ltd. (Shanghai, China) using Illumina sequencing technology. The MEGAX 10.0.5 software was used to execute multiple sequence alignment, and phylogenetic tree of the enriched sequences. The secondary structure and free energy of the enriched sequence were predicted utilizing UNAFold Online Web server.^1^

### Binding properties of aptamers

The binding ability of the aptamers were verified by fluorescence-graphene oxide (GO) assays. Different concentrations (from 0 to 400 μg/ml) of the α-La was allowed to react with 100 nM fluorescence-labeled (5′-FAM) aptamer in 200 μL of binding buffer in the dark for 1 h. Graphene oxide at a 20:1 mass ratio to ssDNA is then added to the mixture and further incubated for 15 min. The fluorescence spectrum of the final mixture at 522 nm was then measured using an F-320 fluorescence spectrometer with excitation at 492 nm. Dissociation constants (Kd) were calculated using GraphPad Prism 9.0 software to characterize the affinity of aptamers for α-La. The equation of Y = Bmax*X/(Kd + X), where Y is the fluorescence intensity of FAM-label aptamer from the supernatant, Bmax corresponds to the maximum relative fluorescence intensity, X is the α-La concentration, and Kd is the dissociation constant. In order to evaluate specificity of the aptamer, a similar experiment was performed. Hundred nanometer of aptamer were incubated separately with 50 μg/ml of other proteins (β-Lg, Cas, BSA, IgG). The fluorescence intensity of the supernatant after incubation was measured and compared with the fluorescence intensity of α-La and blank groups.

### Circular dichroism analysis

The structural changes before and after aptamer binding were analyzed by circular dichroism spectropolarimeter (BRIGHTTIME Chirascan). The aptamers before and after binding were measured in the range of 230–310 nm and background signal was subtracted for 50 μg/ml α-La in binding buffer or binding buffer.

### Molecular modeling studies

To investigate the recognition mechanism between the aptamer and α-La, the Lamarckian genetic algorithm of ZDOCK software was used for molecular docking. The Vienna number of the aptamer was formed through the UNAfold Online Web server, and then, the three-dimensional structure with the best predicted energy was constructed using the RNA composer online tool, and the three-dimensional conformation of the aptamer was obtained by mutating the U-base to the T-base. The protein structure of α-La was obtained from the RCSB PDB databank (PDB ID: 1F6R^2^).

### Colorimetry/fluorescence dual-mode detection for α-lactalbumin

#### Synthesis of BNQDs/CeO_2_

The preparation of BNQDs was carried out according to the previously reported method (29). The BNQDs/CeO_2_ was synthesized applying Zhu et al.’s (30) method with minor modifications. First, the non-porous CeO_2_/Ce(OH)_3_ precursor was obtained at 100^°^C (31). Subsequently, 5 ml of the BNQDs solution was mixed with 100 ml of the 2 mg/ml precursor suspension with continuous stirring, and transferred to an autoclave and kept at 160°C for 12 h. The final product was washed with ultrapure water and dried to obtain BNQDs/CeO_2_.

#### Detection of α-lactalbumin

The aptamer LA-1t was first incubated with BNQDs/CeO_2_ for 20 min at room temperature to obtain BNQDs/CeO_2_@Apt. Then, different concentrations of α-La were added and incubated at 37^°^C for 30 min to fully bind α-La to LA-1t. Thirty millimeter H_2_O_2_ and 1 mM 3,3′,5,5′-Tetramethylbenzidine (TMB) were added at 37^°^C, and after 10 min of color development, the absorbance at 650 nm was measured using a UV-Vis spectrophotometer, and the fluorescence wavelength of all samples was measured using an F-320 fluorescence spectrophotometer (Ex = 492 nm, Em = 522 nm). The procedure was performed in pH = 4.0, 0.2 M acetate buffer.

#### Analytical application to real sample

This developed method was verified to detect α-La in infant amino acid formula powder and milk, were pretreated before analysis. First, a certain amount of α-La standard was added to real samples at concentrations of 2, 1, and 0.1 mg/g (mg/ml), respectively. Simultaneously, unspiked samples of the same concentration were prepared. Then, each of the above two sets of samples were diluted 100 times with PBS, and centrifuged at 8,000 rmp for 20 min to remove fat, collected the subnatant, and subsequently analyzed through the above-mentioned operation process. A similar sample preparation procedure was used when analyzing α-La, using a commercial ELISA kit.

## Results and discussion

### Selection of aptamers for α-lactalbumin

**FIGURE 1:**
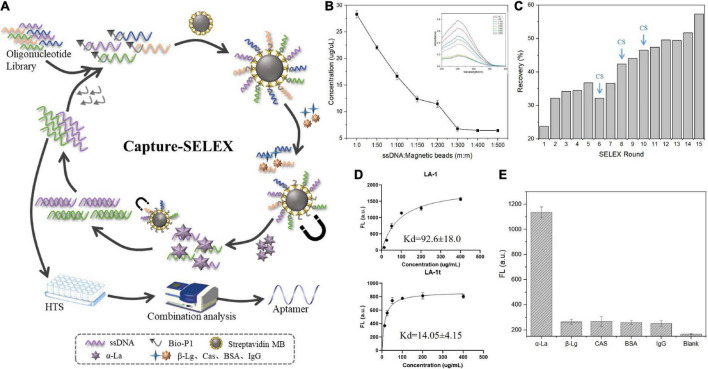
**(A)** Principle of selecting α-lactalbumin (α-La) aptamers using capture-SELEX; **(B)** Optimization of the mass ratio of ssDNA to streptavidin magnetic beads; **(C)** Relative enrichment rate during SELEX for monitoring; **(D)** The binding saturation curves of aptamer LA-1 and LA-1t; **(E)** The specificity of LA-1t.

The schematic diagram of Capture-SELEX is shown in Figure 1A. Instead of immobilizing the target molecule, Capture-SELEX captures oligonucleotides (SELEX library or selected oligonucleotide library) on magnetic beads with a complementary sequence. Magnetic bead-bound components were efficiently separated from other components of the solution using a magnetic field. Before screening, the mass ratio of dsDNA to magnetic beads was determined to be 1:300 (Figure 1B). In total, 15 rounds of Capture-SELEX were executed. To select α-La aptamers with high affinity, the concentration of α-La and the time with α-La were gradually declined during the selection process in this work. Moreover, counter-SELEX was introduced after the 6th round to apply more pressure on selection of highly specific ssDNA sequences. The details are shown in Supplementary Table 1. As well, the concentration of eluted affinity sequences was measured by a micro-UV spectrophotometer to assess the selection process. Results are shown in Figure 1C. During the whole process, even though the screening pressure gradually increased, the recovery rate revealed an upward trend, and the relative decrease in the 6th round may be caused by the reverse screening of other introduced proteins. The recovery rate reaches the maximum at 15 rounds. To ensure sequence abundance, PCR products from 15th ssDNA were analyzed by high-throughput sequencing (HTS).

### Binding identification of α-lactalbumin aptamer candidates

The GO material is an efficient fluorescent acceptor, can quench fluorescence through energy transfer, and it can physically adsorb ssDNA on the GO surface through hydrophobic interactions with bases and π-π stacking interactions. Based on these properties of GO, the affinity and specificity of the aptamers were selected for analysis by fluorescence assay. When no α-La exists, the FAM-labeled aptamer was adsorbed by GO and undergoes FRET to quench its fluorescence signal; while the complex bound to the α-La no longer interacts with GO and this displayed a high fluorescence value. Based on signal changes, the Kd values was calculated.

Sequence alignment of the HTS results, five sequences, with more frequency and less free energy (ΔG), were selected as the candidate aptamers. It can be seen from Supplementary Table 2 that these five candidate aptamers all have a similar conserved sequence GTGCTGCG after the fixed sequence at the 5′ end. By predicting their respective secondary structures by UNAfold (see text foot note 1), it was found that the presence of a consensus sequence made them have similar stem-loop structures at their 5′ end. In addition, since Capture-SELEX is immobilized using complementary sequences to the 3′ end of the aptamer. It should be noted that its immobilized sequence will not participate in its own folding. Based on this it is inferred that the stem-loop structure at the 5′ end is a potential region to achieve binding to α-La. The sequence with the highest affinity among the candidate aptamers was truncated, and only the stem-loop structure containing the consensus sequence was retained. We found that the 30-nt LA-1t after the redundant sequence was removed had about 6-fold higher affinity than the full-length sequence (Figure 1D). Using the same principle, the binding of LA-1t to other negative proteins was tested, and the results illustrated that the aptamer LA-1t demonstrated high specificity for α-La (Figure 1E). In addition, for validation, we designed the base mutant MLA-1t of LA-1t. mutating C3 and A7 to T bases and mutating C24 to T bases. Software predictions showed that the mutation design resulted in any significant change in its secondary structure, losing the original stem-loop structure (Supplementary Figure 1A). Moreover, its binding ability was significantly lost compared to the original sequence (Supplementary Figure 1B), which demonstrated the LA-1t-specific binding ability.

### Study on the binding mechanism between aptamer α-lactalbumin and LA-1t

**FIGURE 2:**
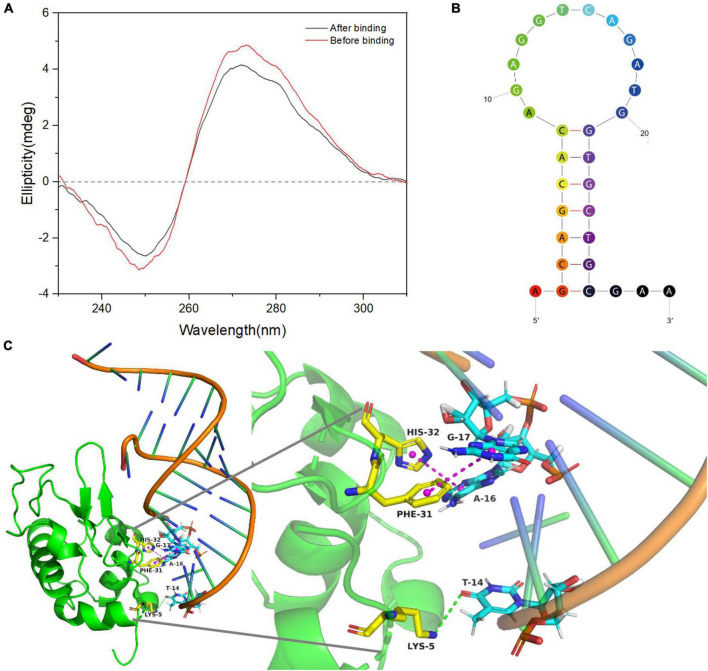
Binding mechanism of α-lactalbumin (α-La) and LA-1t. **(A)** Circular dichroism of LA-1t before and after binding; **(B)** Secondary structure of LA-1t predicted by UNAfold online software; **(C)** The result of molecular docking between aptamer α-La and LA-1t.

Circular dichroism is a derived UV-vis absorption method that can be used to study conformational changes in biomolecules. The CD spectra of the for LA-1t (Figure 2A), sequence exhibited a positive peak at 272 nm and a negative peak at 250 nm characteristic of a known B-type right-handed double helix DNA structure (32). The characteristic peak is mainly caused by the base stacking in the complementary region and the right-handed helical structure. This is consistent with the secondary structure of LA-1t predicted by UNAfold online software, which proves that LA-1t forms a hairpin structure in solution (Figure 2B). When LA-1t combined with α-La, the characteristic absorption peak of LA-1t displayed a slight shift. As well, the CD signal diminished significantly, indicating that the combination of the two had indeed changed the structure of LA-1t. It is speculated that the binding of LA-1t to α-La leads to disruption of the hairpin structure.

The binding mechanism and possible binding sites of the aptamer-protein complex were further investigated by molecular docking. The binding conformation with the best docking score was selected from the best cluster. As presented in Figure 2C, the hydrogen bond and hydrophobic interaction between the aptamer LA-1t, and α-La play a major role in the formation of the complex. The main binding sites of the aptamers are the hydrophobic interactions between A16, G17, and His, Phe, and the hydrogen bond formed between T14 and Lys. These interactions further stabilized the bonding between α-La and LA-1t. Based on the analysis using ZDOCK software, the ZRANK energy between α-La and LA-1t was -155.947 kcal/mol.

### Colorimetry/fluorescence dual-mode detection for α-lactalbumin

**FIGURE 3:**
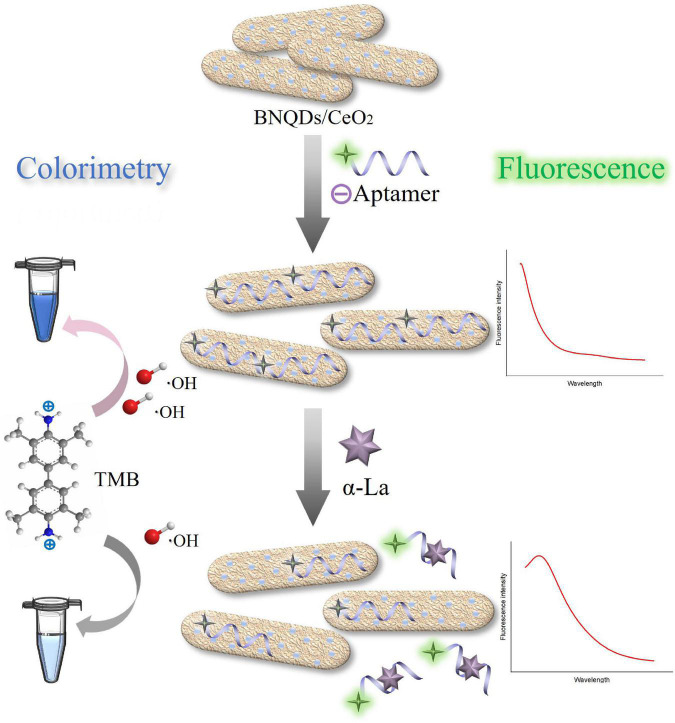
Mechanism of dual-sensing strategy for α-lactalbumin (α-La) detection.

The colorimetry/fluorescence dual-mode sensing strategy to detect α-La is shown in Figure 3, the peroxidase activity of BNQDs/CeO_2_ can catalyze the decomposition of H_2_O_2_ to generate hydroxyl radicals (⋅OH), and oxidize TMB to form blue products (oxTMB). Modified by LA-1t, the BNQDs/CeO_2_ nanozymes increasingly dispersed due to electrostatic interaction, exposing more active sites, the system blue was deepened. And the fluorescence of the labeled FAM on LA-1t was quenched due to FRET with CeO_2_. When α-La was present, LA-1t binds to α-La, thereby dissociating from BNQDs/CeO_2_, in turn resulting in a decline in color signal and recovery of fluorescence, enabling colorimetry/fluorescence detection of α-La-based aptamer regulation strategy. As previously reported, aptamers do not require labeling, and cerium is able to coordinate strongly with the phosphate backbone of nucleotides (33, 34).

#### Characterization of BNQDs/CeO_2_

**FIGURE 4:**
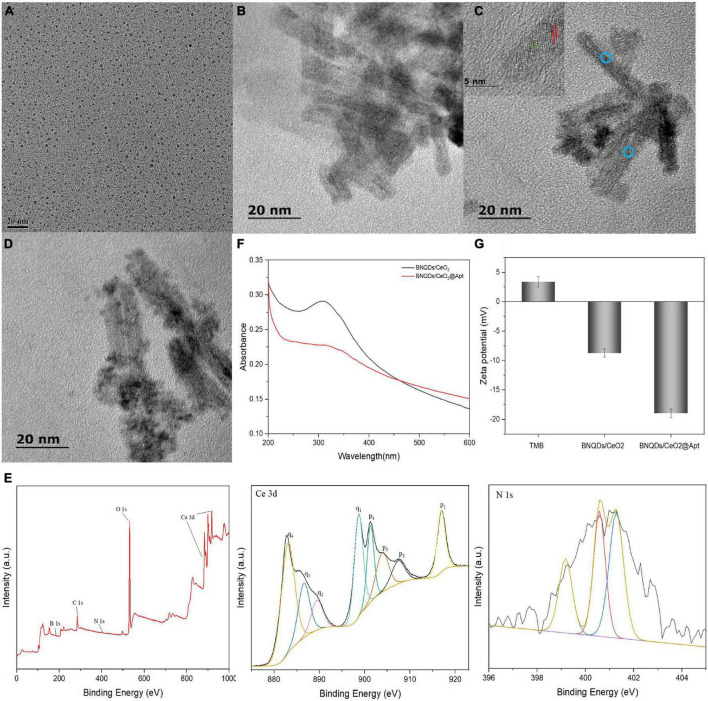
Transmission electron microscopy (TEM) image of **(A)** BNQDs; **(B)** CeO_2_/Ce(OH)_3_ nanorod precursor; **(C)** BNQDs/CeO_2_; **(D)** BNQDs/CeO_2_@Apt; **(E)** XPS survey spectrum of BNQDs/CeO_2_, and high-resolution XPS spectra of Ce 3d, and N 1s; **(F)** UV-Vis absorption spectra of BNQDs/CeO_2_ and BNQDs/CeO_2_@Apt; **(G)** Zeta potential of TMB, BNQDs/CeO_2_, and BNQDs/CeO_2_@Apt.

The morphological structure of the synthesized materials was characterized by transmission electron microscopy (TEM). As illustrated in Figure 4A, the synthesized BNQDs were spherical with uniform size, and their average particle size was about 2 nm. In Figure 4B, the CeO_2_/Ce(OH)_3_ precursor is a uniformly dispersed non-porous rod-like morphology with a length of 30–40 nm and a diameter of about 5 nm. As shown in Figure 4C, BNQDs/CeO_2_ can find some dark spots uniformly distributed on the surface of the nanorods, and the lattice fringes of CeO_2_ and BNQDs can be clearly observed, further revealing their combination (35).

X-ray photoelectron spectroscopy (XPS) was used to further confirm the presence of Ce, B, N, O, and C elements (Figure 4E). The four pairs of spin-orbit peaks in the Ce 3d spectrum demonstrate the complex valence state of Ce, reflecting the existence of oxygen vacancies on the surface of BNQDs/CeO_2_. Moreover, the peak at about 531.9 eV of the O 1 s XPS spectrum belongs to surface adsorbed oxygen species (Oα), which is related to the presence of oxygen vacancies. The three peaks are located at 399.1, 400.58, and 401.28 eV in the N 1 s spectrum, which belong to the N-B, N-C and N-H bonds, respectively. Moreover, the B 1 s spectrum is fitted to two peaks with binding energies at 190.9 and 192.08 eV that correspond to the B-N and B-O bonds, respectively (Supplementary Figure 3). In summary, the BNQDs/CeO_2_ was synthesized successfully.

#### Validation of peroxidase-mimic activity and fluorescence quenching properties of BNQDs/CeO_2_

The peroxidase-mimic activity enhancement and fluorescence quenching events occurred after the FAM-aptamer adsorption. Figure 4D is the TEM image of aptamer-modified BNQDs/CeO_2_. It can be seen that the surface of the rod-shaped BNQDs/CeO_2_ uniformly adsorbs some nano-scale dark spots, and is more dispersed than the pure BNQDs/CeO_2_. As presented in Figure 4F, after aptamer-modified BNQDs/CeO_2_, a new peak appeared in the UV-Vis spectrum at about 260 nm, corresponding to the characteristic absorption peak of single-stranded DNA. In addition, the surface charge states of TMB, BNQDs/CeO_2_ and BNQDs/CeO_2_@Apt were investigated using zeta potential (Figure 4G). TMB exhibits a positive charge due to the presence of amino groups. Conversely, the potential value of BNQDs/CeO_2_ were negative, and the potential value of BNQDs/CeO_2_@Apt was more negative after modification with aptamer, which may be due to the negatively charged phosphate backbone of the aptamer. These indicate that the aptamer LA-1t successfully binds with BNQDs/CeO_2_.

The peroxidase-like activity investigation was performed by H_2_O_2_ oxidation of TMB chromogenically. From Supplementary Figure 2A, it can be seen that the individual substrates TMB and H_2_O_2_ did not appear to have obvious absorption peaks (curve a). However, a visible peak at 650 nm (curve d) appeared after the addition of BNQDs/CeO_2_, and the intensity was higher than that of the BNQDs and CeO_2_/Ce(OH)_3_ precursor (curve b and c). When aptamer-modified BNQDs/CeO_2_, the peroxidase-mimicking activity was enhanced approximately 1-fold (curve f), and the color of the solution system was become even bluer. When α-La was added to the reaction system, the intensity of the absorption peak decreased significantly. This indicates that the aptamer binds to α-La, thereby desorbing from the surface of BNQDs/CeO_2_. By calculating the initial reaction velocities of TMB and H_2_O_2_, we obtained Michaelis-Menten curves (Supplementary Figures 3A,B) and key enzyme kinetic parameters such as maximum initial velocity (*V*max) and Michaelis-Menten constant (Km). The relevant values are summarized in Supplementary Table 3. Results show that compared with BNQDs/CeO_2_, BNQDs/CeO_2_@Apt has a higher affinity for two substrates and a faster rate. This means that the introduction of aptamers improves the catalytic activity and catalytic rate of BNQDs/CeO_2_. This may be due to the introduction of negatively charged aptamers to make BNQDs/CeO_2_ more dispersed, exposing more active sites.

In the fluorescence mode, the fluorescence intensity of FAM was recorded as the sensing signal. In Supplementary Figure 2B, LA-1t emitted a strong fluorescent signal at 522 nm (curve f). However, nearly half of the fluorescence intensity was quenched after the addition of BNQDs/CeO_2_ (curve d), indicating that FRET occurred after successful adsorption of aptamers. After the introduction of α-La, the fluorescence intensity recovered significantly (curve e). And the BNQDs/CeO_2_ material itself has no fluorescence (curves a–c). The above results confirmed the feasibility of developed colorimetry/fluorescence dual-mode sensing strategy based on aptamer-regulated BNQDs/CeO_2_ for the α-La detection.

#### Optimization of reaction conditions

The effects of α-La aptamer concentration, temperature, pH and color development time were investigated so as to optimize the dual-mode assay for optimum signal. Supplementary Figure 4A exhibits that at low concentrations, the catalytic activity was gradually enhanced as the increased of aptamer concentration, and the fluorescence intensity no longer changed significantly. However, when the concentration was greater than 0.5 μM, the absorbance remains stabilized, and the fluorescence intensity increased significantly, indicating that the aptamer adsorbed on the surface of BNQDs/CeO_2_ has reached a saturation state. Additionally, we found that the temperature had little effect on the two analytical modes (Supplementary Figure 4B), indicating that BNQDs/CeO_2_ has an excellent stability. To be consistent with the binding conditions, 37^°^C was chosen as the reaction temperature. As can be seen in Supplementary Figure 4C, within 60 min of adding the chromogenic substrate, the absorbance gradually increased, so the time when the absorbance reached about one was selected as the fixed time, and the time was 10 min. Furthermore, exhibited the strongest catalytic activity at a pH of 4.0 (Supplementary Figure 4D).

#### The performance analysis of the dual-mode sensing strategy for α-lactalbumin

**FIGURE 5:**
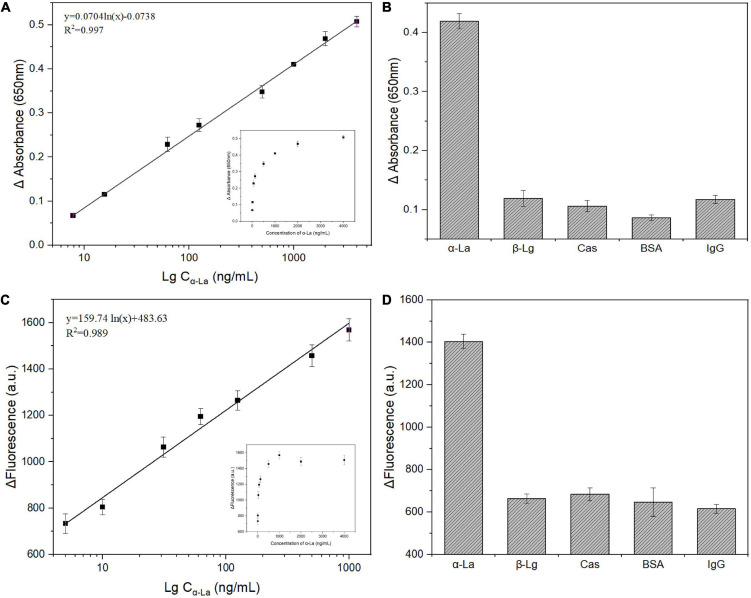
Linear calibration plot between absorbance **(A)** and fluorescence intensity **(C)** versus the logarithm of α-lactalbumin (α-La) concentrations; Selectivity of proposed colorimetric **(B)** and fluorescence **(D)** strategy for α-La detection (1 μg/ml) alone or coexisting with 5 μg/ml of other interferences.

Under optimal conditions, different concentrations of α-La were detected based on this dual-mode strategy. Since the LA-1t were desorbed from BNQDs/CeO_2_ and bound to α-La with higher affinity, the catalytic activity decreased and the fluorescence increased. As shown in Figure 5A, the difference in absorbance (ΔA = A0–A) has a good linear relationship with the logarithm of the α-La concentrations, A0 was the absorbance without adding α-La. The corresponding regression equation was y = 0.0704 ln (x)–0.0738 (*R*^2^ = 0.997), while the limit of detection (LOD, S/N = 3) was 3.32 ng/ml. In fluorescence sensing mode, the difference in fluorescence intensity (ΔF = F-F0) gradually increased with the increase of α-La concentration (Figure 5C), F0 was the fluorescence intensity without adding α-La. When the concentration of α-La increased to 1 μg/ml, the relative fluorescence intensity was linearly related, and the linear regression equation was y = 159.74 ln (x) + 483.63 (*R*^2^ = 0.989). Furthermore, LOD of the fluorescence method was 0.71 ng/ml. After statistical analysis, there was no significant difference (*P* > 0.05) between the two methods. Such detection limits are significantly improved compared to other commonly used approaches or comparable to many α-La complex detection strategies (Table 1).

**TABLE 1:** Comparison of the developed method with other different detection methods for α-lactalbumin (α-La) determination.

Methods	Sensing platform	Detection range	LOD	References
RP-HPLC	-	0.01–5.0 g/L	8 mg/L	(19)
LC-MRM/MS	Peptide fragment	0.97–31.25 μg/ml	0.39 μg/ml	(36)
Colorimetric	Goldmag-mAbs	2.33–127.1 ng/ml	17.2 ng/ml	(37)
FLISA	CdSe/ZnS QDs-mAbs	0.1–1,000 ng/ml	0.1 ng/ml	(12)
Immunochromatographic strip	Colloidal gold-mAbs	-	10 μg/ml	(38)
Electrochemical	SWCNT	20–80 ng/ml	9.7 ng/ml	(39)
Colorimetric/fluorescence	BNQDs/CeO_2_@Apt	5–4,000 ng/ml	3.32 ng/ml, 0.71 ng/ml	This work

The selectivity of this dual-mode strategy was assessed by examining the color and fluorescence intensity changes of solutions in the presence of β-Lg, Cas, BSA, and Ig-G. As shown in Figures 5B,D, the presence of even 5-fold excess (5 μg/ml) of the control target causes insignificant changes in color and fluorescence intensity in solutions. In contrast, the presence of low concentrations of α-La (1 μg/ml) resulted in a vitally increase in the signal, indicating that the colorimetry/fluorescence dual-mode sensing strategy that we constructed was successfully applied to the high-sensitivity and selectivity for α-La detection.

#### Determination of α-lactalbumin in real samples

The milk and infant amino acid formula powder spiked with different concentrations of α-La and samples without standard were used as matrices to test the applicability of this colorimetry/fluorescence dual-mode strategy toward α-La detection. The difference in absorbance and fluorescence intensity between the two groups of samples were used as the analysis index. As illustrated in Table 2, the spiked recoveries were 95.81–115.37%, and the relative standard deviations (RSD) were in the 2.12–6.65% range. Commercial ELISA kit recoveries ranged from 94.27–104.31%, in agreement with the results of our developed sensing strategy, confirming that the proposed BNQDs/CeO_2_@Apt-based colorimetry/fluorescence dual-mode sensing had great potential to detect α-La in real samples.

**TABLE 2:** Determination of α-lactalbumin in spiked food extract.

Sample	Spiked α -La (mg/ml, mg/g)	This work (colorimetry/fluorescence)			Commercial ELISA kits		
		Found (mg/ml, mg/g)	Recovery (%)	RSD (%)	Found (mg/ml, mg/g)	Recovery (%)	RSD (%)
Milk	0.1	0.11/0.12	113.09/115.37	3.74/5.72	0.10	104.25	2.73
	1	1.06/1.09	105.73/108.71	5.86/6.65	1.07	107.31	1.43
	2	2.15/2.04	107.55/102.16	2.12/5.48	2.00	100.50	1.92
Milk power	0.1	0.11/0.10	107.56/101.47	2.12/4.91	0.10	103.11	3.40
	1	1.02/1.05	101.98/104.80	4.99/5.04	0.94	94.27	1.66
	2	1.96/1.92	97.85/95.81	3.41/5.33	1.99	99.68	1.42

## Conclusion

In the present study, we successfully selected aptamers with high affinity and specificity for the milk allergen α-La by 15 rounds of capture-SELEX. The hairpin structure of the consensus sequence was preserved, and LA-1t with only 30 bases was obtained, with the Kd of 14.05 ± 4.15 nM, which was six times higher than the full-length sequence in affinity. The possible recognition mechanism of aptamer LA-1t, and α-La was studied using CD, and molecular simulation technology. Furthermore, based on the aptamer regulated BNQDs/CeO_2_ system, a colorimetry/fluorescence dual-mode sensing strategy was developed for highly selective and sensitive α-La detection. This colorimetry/fluorescence dual-mode sensing strategy displayed sensitive recognition for α-La in a linear range of 5-4,000 ng/ml with the LOD was 3.32 and 0.71 ng/ml, respectively. Meanwhile, the dual-mode sensing strategy performs well in the detection of α-La in spiked real samples with good stability. This dual-mode DNA sensor could be applied to wider food allergen risk management decisions in food manufacturing.

## Data availability statement

The raw data supporting the conclusions of this article will be made available by the authors, without undue reservation.

## Author contributions

RL: data curation, methodology, and writing–original draft. FZ: investigation, validation, and writing–review and editing. MS: investigation, formal analysis, and methodology. YS: investigation, validation, and supervision. XW: project administration, resources conceptualization, and writing–review and editing. All authors contributed to the article and approved the submitted version.

## Footnotes

1
http://www.unafold.org/

2
https://www.rcsb.org/
