# Supplementary material for: In vitro selection and optimization of high-affinity aptamer for milk allergen α-lactalbumin and its application in dual-mode detection
Source: Front Nutr. 2022 Oct 4;9:1005230. doi: 10.3389/fnut.2022.1005230 (PMC9577226; doi:10.3389/fnut.2022.1005230)
Supplement: Supplementary file 1 [file Presentation_1.pdf]

Supplementary Material

1 Supplementary Figures and Tables

1.1 Supplementary Tables Figures

Table S1. Capture-SELEX conditions

SELEX Round	ssDNA Added (pmol)	Time (min)	Counter SELEX	Recovery
1	1000	120	-	23.73%
2	100	120	-	32.14%
3	100	120	-	34.19%
4	100	100	-	34.47%
5	100	100	-	36.78%
6	100	100	β-Lg、Cas、BSA、IgG (50ug/mL)	32.14%
7	100	80	-	36.59%
8	100	80	β-Lg、Cas、BSA、IgG (50ug/mL)	42.37%
9	100	80	-	44.06%
10	100	60	β-Lg、Cas、BSA、IgG (50ug/mL)	46.50%
11	100	60	-	47.39%

12	100	60	-	49.56%
13	100	45	-	49.42%
14	100	45	-	51.66%
15	100	45	-	57.32%

Table S2. Candidates aptamers chosen from sequence families.

Aptamer	Sequence (5’-3’)	Kd (nM)
LA-1	AGCAGCACAGAGGTCAGATGGTGCTGCGAACTTAACGCAAGATAGGCTGGACGCGAGTCCCCTATGCGTGCTACCGTGAA	92.6±18.0
LA-2	AGCAGCACAGAGGTCAGATGGGCTGCGAACAGTTGCATATGGCGCGACTCTAGGCGGGTCCCTATGCGTGCTACCGTGAA	101.3±21.3
LA-9	AGCAGCACAGAGGTCAGATGCTGGGCTGCGAATGACGAATAAATACGCCGTTTCAGACGGCCTATGCGTGCTACCGTGAA	253.0±32.2
LA-10	AGCAGCACAGAGGTCAGATGTAGTGCTGCGAAGTTGGCTCCGGCGGCGTCAGTCTAGCCGCCTATGCGTGCTACCGTGAA	167.6±28.9
LA-11	AGCAGCACAGAGGTCAGATGTGGGTGCTGCGAAAGGACGGCATGGGGTCCAAAAGACCCCCCTATGCGTGCTACCGTGAA	174.7±28.3
LA-1t	AGCAGCACAGAGGTCAGATGGTGCTGCGAA	14.05±4.15
MLA-1t	AGTAGCTCAGAGGTCAGATGGTGAT GCGAA	-

Table S3. Comparison of Km and Vmax of BNQDs/CeO_2 and BNQDs/CeO_2@Apt.

Catalyst	Substrate	Km (mM)	Vmax(10−8M·s−1)
BNQDs/CeO2@Apt	H2O2	5.59	6.72
	TMB	0.55	22.23
BNQDs/CeO2	H2O2	11.52	5.39
	TMB	0.73	20.40

1.2 Supplementary Figures

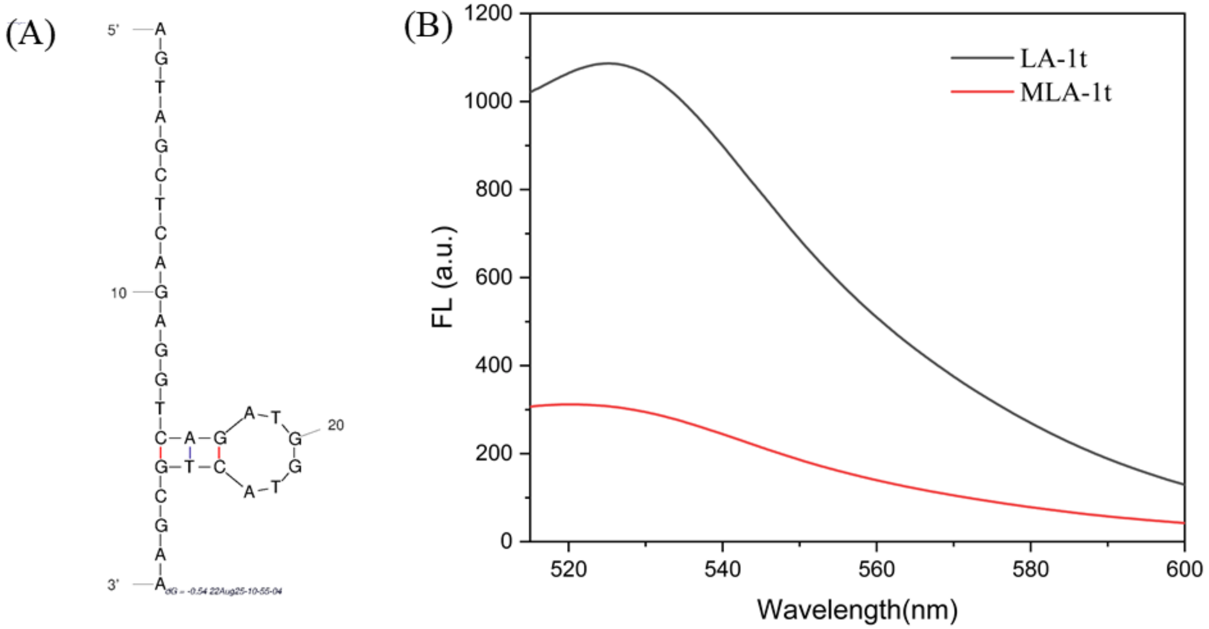

Supplementary Figure 1. (A) Secondary structure of the mutant sequence MLA-1t. (B)Comparison of α-lactalbumin binding ability between LA-1t and the mutant sequence MLA-1t.

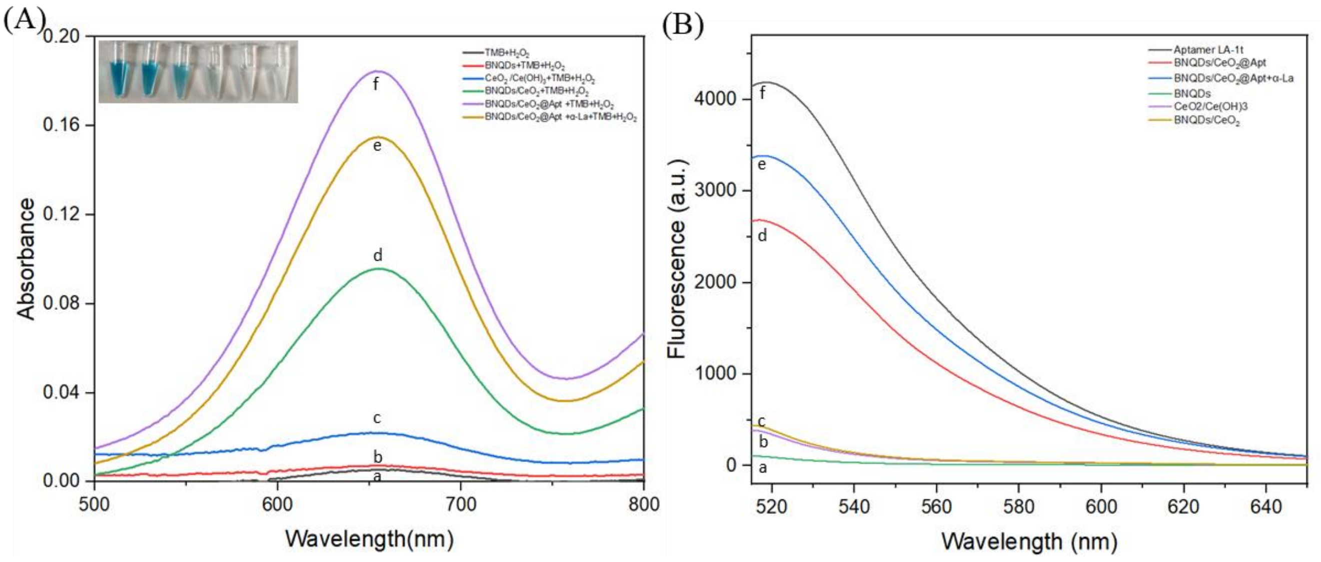

Supplementary Figure 2. (A) UV-vis absorption spectra of (a) TMB+H_2O_2; (b) BNQDs+TMB+H_2O_2; (c) CeO_2/$Ce(OH)_3$+TMB+H_2O_2; (d) BNQDs/CeO_2+TMB+H_2O_2; (e) α-La (1 μg/mL)+BNQDs/CeO_2@Apt+TMB+H_2O_2; (f) BNQDs/CeO_2@Apt+TMB+H_2O_2. (B) Fluorescence spectroscopy of (a) BNQDs; (b) CeO_2/$Ce(OH)_3$; (c) BNQDs/CeO_2; (d) BNQDs/CeO_2@Apt; (e) α-La (1 μg/mL)+BNQDs/CeO_2@Apt; (f) Aptamer LA-1t.

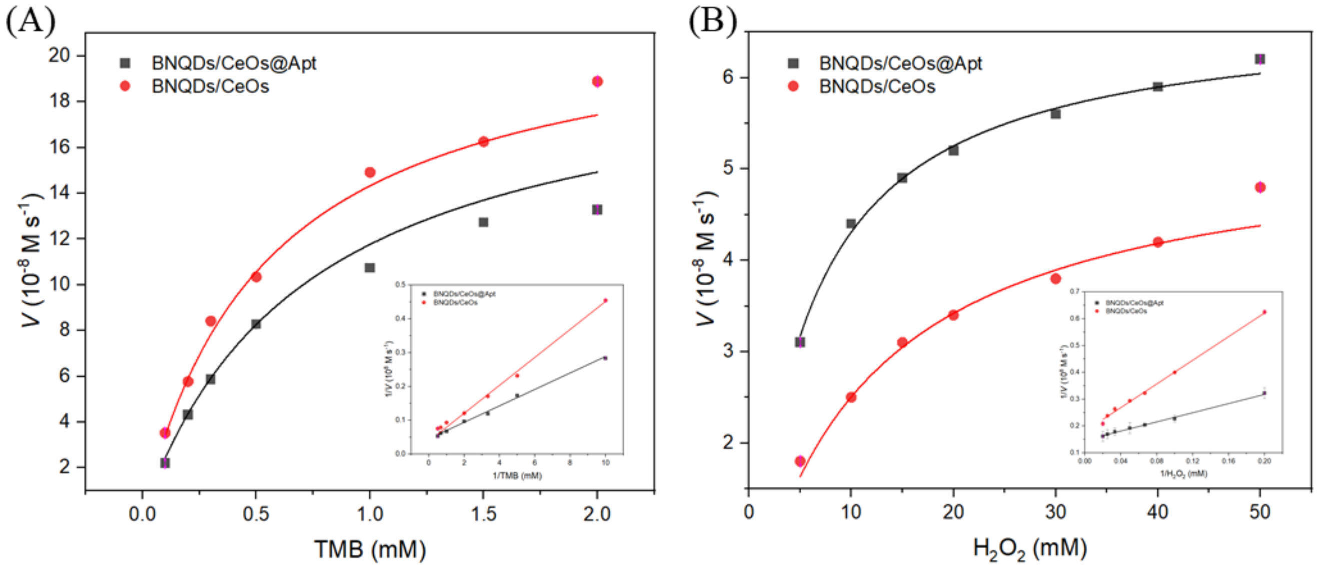

Supplementary Figure 3. The Michaelis-Menten curves and the corresponding Lineweaver-Burk double reciprocal plots of BNQDs/CeO_2 and BNQDs/CeO_2@Apt for (A) TMB and (B) H_2O_2.

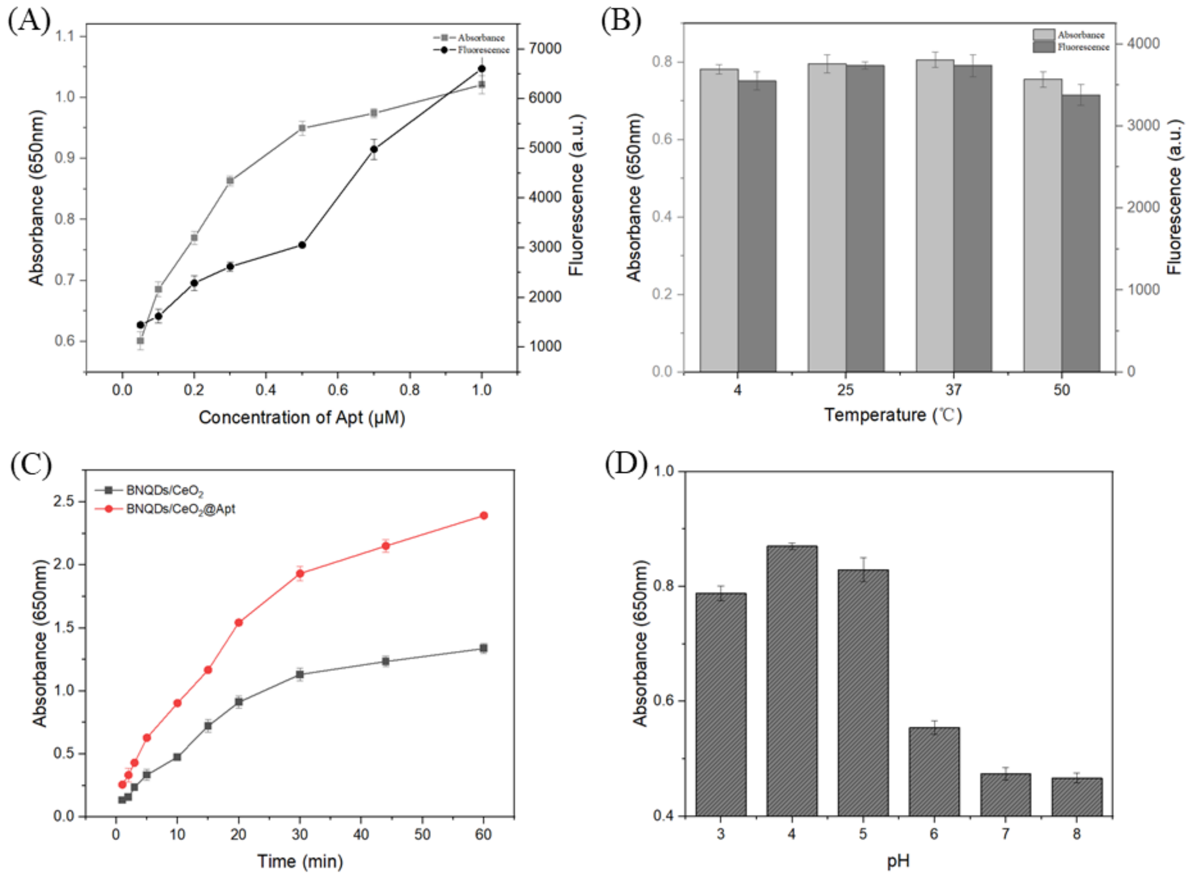

Supplementary Figure 4. Optimization of reaction parameters. (A) Aptamer concentrations; (B) Working temperature; (C-D) Catalytic reaction time and pH.
